# Diagnostic efficacy of fecal markers combined with alarm symptoms in distinguishing functional and organic abdominal pain in children

**DOI:** 10.3389/fped.2025.1707990

**Published:** 2026-01-06

**Authors:** Junhong Liu, Bin Wu, Bihong Ma, Xiaoyan Zhang

**Affiliations:** 1 Department of Pediatrics, The First Affiliated Hospital of Fujian Medical University, Fuzhou, China; 2 Department of Epidemiology and Health Statistics, School of Public Health, Fujian Medical University, Fuzhou, China

**Keywords:** chronic abdominal pain, functional abdominal pain, fecal calprotectin, fecal occult blood test, ROC curve

## Abstract

**Objective:**

This study assessed the diagnostic potential of fecal markers, calprotectin (FC), and occult blood (OB), in conjunction with alarm symptoms and blood markers, to differentiate functional abdominal pain (FAP) from organic disease in children.

**Methods:**

We conducted a retrospective review of children with chronic abdominal pain who presented between April 2017 and April 2020, which included 347 classified as FAP and 224 classified as organic disease. Children with constipation in the functional group were included, and a slight elevation in FC in that group was considered non-inflammatory. FC interpretation included age/sex norms as opposed to percentiles, utilizing the cutoff of 60 µg/g, with justification given by the European Society for Paediatric Gastroenterology, Hepatology and Nutrition diagnostic criteria (*Journal of Pediatric Gastroenterology and Nutrition*, 2021; 72: 617–640). We also analyzed a second threshold of 100 µg/g. Families were sent instructions on how to do the OB test to reduce the false-positive rate. We utilized receiver operating characteristic curves to quantify diagnostic performance.

**Results:**

The organic disease had significantly greater alarm symptoms and abnormal fecal and blood indices compared with FAP (*P* < 0.05). Alarm symptoms with fecal markers produced diagnostic accuracy compared with the six strategies we tested (AUC = 0.841, sensitivity = 90.6%, specificity = 70.3%). Both of these scores exceeded either of the markers individually.

**Conclusion:**

The combination of alarm symptoms with fecal FC and OB tests provides a sensitive and specific non-invasive strategy for the differentiation of organic from FAP in children. The mild elevation of FC is likely indicative of functional constipation rather than inflammation, and attention should be given to diet and substances that affect OB outcome, specifically in children.

## 1 Introduction

Chronic abdominal pain (CAP) is a diverse collection of clinical syndromes with a variety of causes and therapeutic possibilities. CAP is a reasonable common challenge for children and adolescents, with epidemiological study prevalence ranging from 0.3% to 19% in developed countries and as high as 41% in developing regions (1–3). The distinction between organic and functional disease provides the basis for diagnosis and management through targeted treatment to that specific diagnosis (4).

Endoscopy and histopathology remain the gold standards for differentiating organic gastrointestinal disorders and chronic functional abdominal pain (FAP). Although both endoscopic procedures are invasive procedures, they are often costly, with limited tolerance in children, and may not be a feasible choice for an initial screening option (5). In the last decade, fecal calprotectin (FC) has been a reliable non-invasive biomarker of intestinal mucosal inflammation. In practice, FC is often tested in combination with classic blood indices to enhance diagnoses (6).

Normal concentrations are age dependent, with total FC concentration highest in infancy and then declining toward adolescence and adulthood (7, 8). This study selected a single cutoff (60 µg/g) to maintain diagnostic consistency based upon the European Society for Paediatric Gastroenterology, Hepatology and Nutrition (ESPGHAN) position paper (9). This cutoff allows for an additional point of comparability and may decrease specificity in younger children and benign conditions. Previous studies showed that mildly elevated FC concentrations (60–100 µg/g) can be seen in functional constipation and other non-inflammatory causes, while a more markedly elevated FC (>150–200 µg/g) is more consistent with organic gastrointestinal disease (10). Thus, utilizing multiple cutoff thresholds can be more accurate for diagnosis.

Functional abdominal pain disorders (FAPD) would differ from organic causes, including inflammatory bowel disease (IBD), peptic ulcer, or colorectal polyps, with no demonstrable mucosal inflammation, despite some overlapping clinical symptoms. Fecal occult blood (OB) testing is another non-invasive test used in practice to identify gastrointestinal bleeding, with false positives from diet or medications (11). Combination fecal hemoglobin and calprotectin appears to show differences as well for being able to differentiate functional from organic bowel disease (10).

To establish diagnostic certainty for this study, we considered non-specific intestinal inflammation as organic gastrointestinal disease when there was evidence of mucosal erythema, edema, or erosions demonstrated on both endoscopic and histopathologic examination. Differently, children with FAP not otherwise specified (FAP-NOS) showed completely normal endoscopic and histologic examination findings, confirming that no organic pathology existed.

Therefore, this study evaluated the diagnostic utility in differentiating functional from organic abdominal pain in the pediatric population using fecal markers in conjunction with alarm symptoms as a cost-effective and non-invasive diagnostic approach for the pediatric clinical setting.

## 2 Method

### 2.1 General information and diagnostic criteria

We retrospectively reviewed the medical records for children with chronic abdominal pain admitted to the Department of Pediatrics at the First Affiliated Hospital, Fujian Medical University, between April 2017 and April 2020. Eligible subjects were aged from 2 to 14 years, had chronic abdominal pain with a duration of at least 2 months (Rome III chronic abdominal pain) before admission, and had complete clinical data available. Symptoms had to be classified as occurring >1 day per week or >4 days a month.

Subjects were excluded from the study if they had a diagnosis of any cancer (current and past), had established immunodeficiency or other systemic diseases, had taken non-steroidal anti-inflammatory drugs or glucocorticoids prior to the completion of their examination, or had missing data or were lost to follow-up. The patients were categorized into the FAPD group or the organic disease group by their final clinical diagnosis. The diagnostic work-up included clinical assessment, laboratory data, imaging, and an endoscopy to rule out organic gastrointestinal diseases, such as IBD, peptic ulcer disease, and gallbladder disease.

To ensure diagnostic accuracy, non-specific intestinal inflammation was diagnosed as organic disease only when mucosal abnormalities were confirmed by both endoscopy and histopathology, even when no specific etiology was identified.

The identification of non-specific intestinal inflammation, defined as an “organic disease” that had been confirmed by endoscopic and histopathologic evidence of erythema, edema, and/or erosions of the mucosa (with or without a specific etiology), was based on common pediatric gastroenterology practice. For all patients diagnosed with FAPD, upper and/or lower endoscopy was performed if alarm symptoms were present (e.g., unintentional weight loss) or if the FC was ≥60 µg/g, ensuring that organic gastrointestinal disease was ruled out before confirming a clinical diagnosis.

In total, 69/347 children in the FAPD group underwent upper and/or lower endoscopic evaluation to exclude organic gastrointestinal diseases.

The Rome IV criteria were applied to characterize FAPD subgroups, including functional dyspepsia (FD), irritable bowel syndrome, abdominal migraine, and FAP-NOS. The study was approved by our hospital's ethics committee (Ethics Batch No. [2015]084), and written informed consent was obtained from the parent or guardian.

Normal FC reflects child age, with infants or younger children typically having higher levels. A single cut point as the threshold of 60 µg/g was recommended for consistency with other pediatric studies (9). Secondary analyses also stratified patients by age to assess for any differential diagnostic performance. The study included children with functional constipation since it was possible for mild FC elevation to occur in this subgroup, which was taken into consideration when interpreting results and was also stated as a limitation of the study.

This study measured FC only, and no serum calreticulin testing was performed.

### 2.2 Clinical data and laboratory measures

Demographic information was collected on the child: age and gender, time of first visit, clinical diagnosis, and presence of alarm symptoms, which consisted of abdominal pain, fever of unknown origin, diarrhea, constipation, vomiting, bloody stool, abdominal distension, perianal disease, growth retardation, weight loss, delayed puberty, oral ulcer, and family history. In addition, arthritis and arthralgia were documented as extraintestinal manifestations of organic gastrointestinal disease.

Data from laboratory testing included blood indices—white blood cell count (WBC), platelet count (PLT), C-reactive protein (CRP), and erythrocyte sedimentation rate (ESR)—and fecal testing parameters (FC and OB). Prior to collecting the OB sample, parents were given information on dietary restrictions and medication restrictions before performing the test to reduce the variation of false-positive results, advising them to avoid red meat, horseradish, broccoli, non-steroidal anti-inflammatory drugs, and high-dose vitamin C for 72 h prior to the sample collection.

### 2.3 Combination and analysis of diagnostic strategies

The diagnostic performance was evaluated separately, for each indicator, as well as for six predefined combined strategies [i.e., alarm symptoms only; combined blood indices (WBC, PLT, CRP, ESR); combined fecal indices (FC, OB); alarm symptoms + blood indices; alarm symptoms + fecal indices; alarm symptoms + blood and fecal indices]. A positive result was defined in any strategy as one or more alarm symptoms or one or more abnormal laboratory indicators. For FC, the primary analysis used the uniform cutoff of 60 μg/g; a separate age-stratified analysis, which took into account physiological differences in normal values, was carried out.

**Figure 1:**
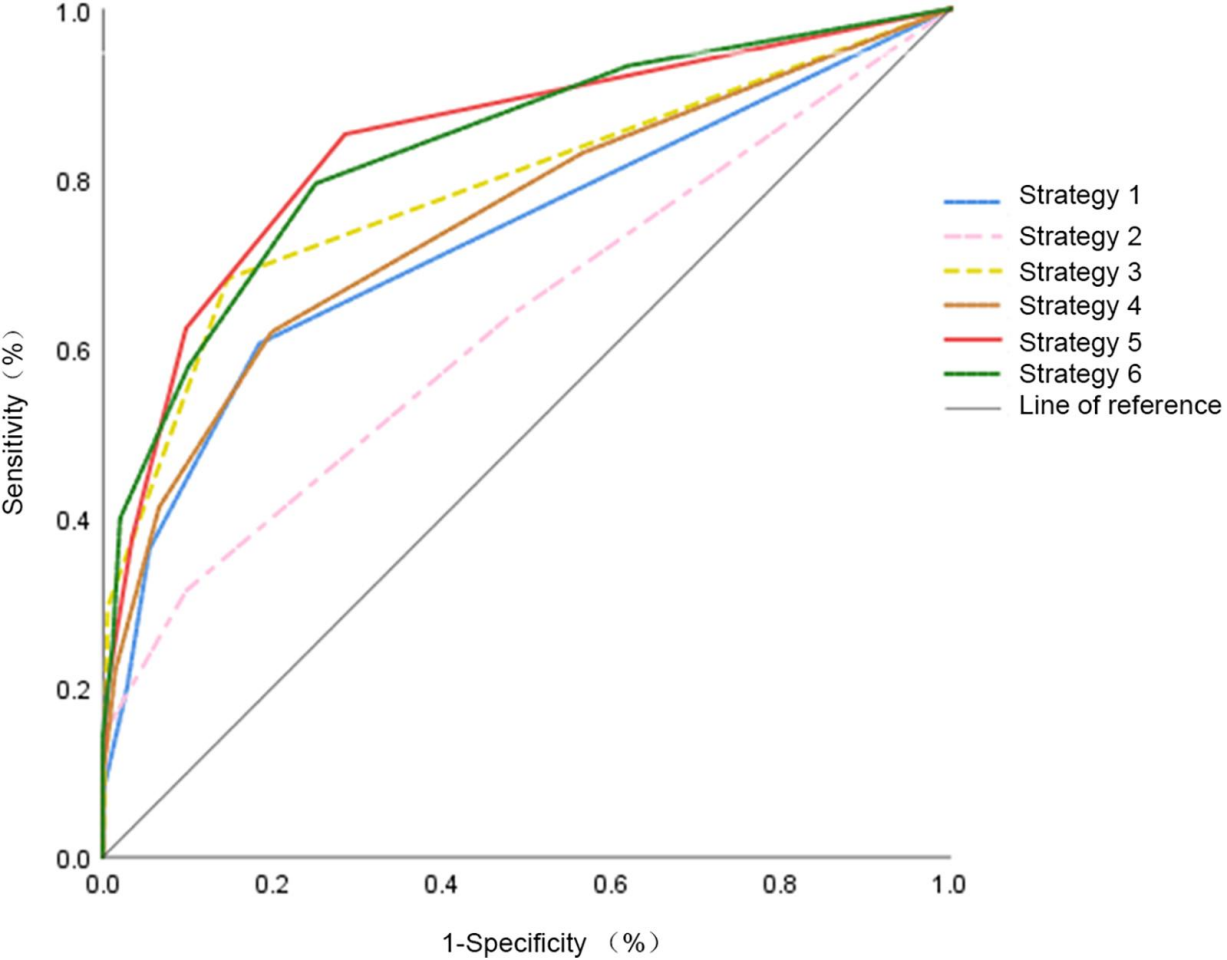
ROC curves for each co-diagnostic indicator. This figure shows the ROC curves of the co-diagnostic parameters, indicating their combined diagnostic value in differentiating organic diseases and FAPD.

To ensure clarity in the ROC analyses presented in Figure 1, the six predefined diagnostic strategies were defined as follows:

**Strategy 1—alarm symptoms only**
A positive result was defined as the presence of one or more alarm symptoms.
**Strategy 2—blood indicators only**
A positive finding required at least one abnormal blood inflammatory marker (WBC, PLT, CRP, or ESR).
**Strategy 3—fecal indicators only**
A positive result was recorded when either FC (FC ≥ 60 µg/g) or fecal OB was abnormal.
**Strategy 4—alarm symptoms + blood indicators**
A strategy was considered positive if the child exhibited at least one alarm symptom and/or at least one abnormal blood marker.
**Strategy 5—alarm symptoms + fecal indicators**
A positive classification required the presence of one or more alarm symptoms and/or abnormal FC or OB.
*(This strategy demonstrated the highest diagnostic accuracy.)*
**Strategy 6—alarm symptoms + blood + fecal indicators**
A positive result was defined as the presence of any alarm symptom/or any abnormal blood marker/or any abnormal fecal marker.

These definitions correspond to the diagnostic schemes used in the ROC curve comparisons shown in Figure 1.

### 2.4 Statistics

Statistical analyses were conducted using SPSS version 20.0 (IBM Corp., Armonk, NY, USA). Continuous variables were expressed as mean ± SD, and independent-sample *t*-tests were used to examine between-group differences. Categorical variables were presented as counts and percentages compared using chi-squared or Fisher's exact tests. The main analysis evaluated the diagnostic performance of each indicator and the combined strategy in distinguishing between FAP and organic abdominal pain in children. Sub-group analysis was carried out to test FC diagnostic accuracy in different age groups and patients with functional constipation. Receiver operating characteristic (ROC) curves were constructed for each indicator and combined strategy, and the area under the curve (AUC), sensitivity, specificity, positive predictive value (PPV), and negative predictive value were determined for each area. A *P*-value of <0.05 was considered statistically significant.

## 3 Results

### 3.1 Epidemiological features of the study population

Through medical record review, 729 children with chronic abdominal pain were initially identified. We excluded 8 children with immunodeficiency, tumor, and other basic diseases were excluded, 14 children treated with non-steroidal drugs or glucocorticoids prior to examination, and 136 children lost to follow-up with incomplete clinical information. In total, 571 children were included in the study, with 224 organic disease cases and 347 FAPD cases. Gender and mean age for comparison between groups are presented in Table 1. The number of boys was significantly higher than girls, with the predominant age of both groups being 7–12 years, accounting for 73% and 56% of the total cases in each group, respectively.

**Table 1:** Comparison of basic information of patients in two groups.

Groups	Gender (*n*, %)		Age ($\bar{x}\pm s$, years)
	Male	Female	
Organic disease(*n* = 224)	150 (67.0)	74 (33.0)	9.1 ± 2.7
FAPD (*n* = 347)	203 (58.5)	144 (41.5)	7.8 ± 2.6
*t*/*χ*^2^	4.13		5.66
*P*-value	**0.042**		**<0.01**

FAPD, functional abdominal pain disorder.

*P*-value of <0.05 was considered statistically significant.

When stratified by age, the FC positivity rate (>60 μg/g) in the organic disease group was 68.2% in 2–6-year-olds, 58.4% in 7–12-year-olds, and 54.5% in 13–14-year-olds, whereas in the FAPD group, it was 21.7%, 14.9%, and 10.5%, respectively (data not shown in tables).

The median FC level was 95 μg/g (IQR: 60–180) in the organic disease group vs. 35 μg/g (IQR: 20–60) in the FAPD group. The mean FC was significantly higher in organic causes (*P* < 0.001).

Among the 224 organic disease cases, diagnostic procedures included electron gastroscopy in 179 cases, enteroscopy with mucosal biopsy in 127, small bowel magnetic resonance imaging in 84, and capsule endoscopy in 25.

In the FAPD cohort, 76 patients had FD, 141 with irritable bowel syndrome (IBS), 23 with abdominal migraine, and 107 with FAP not otherwise specified (FAP-NOS).

### 3.2 Comparing alarm symptoms between groups

The distribution of alarm symptoms is presented in Table 2. Of the 571 patients, 227 (40%) had positive alarm symptoms, with 158 (71%) in the organic disease group and 69 (20%) in the FAPD group. Between-group differences were statistically significant (*P*< 0.001). The most common alarm symptom was vomiting (14.5%), followed by weight loss (14%), positive family history (Helicobacter pylori (HP) infection/gastric ulcer/IBD, 13%), diarrhea (10.5%), and bloody stool (9.5%). Statistical analyses indicated significant differences in vomiting, weight loss, positive family history, diarrhea, bloody stool, fever, abdominal distension, perianal disease, growth retardation, and oral ulcer between groups (*P* < 0.05). Arthritis or arthralgia was identified in 11 children (4.9%) in the organic disease group and two children (0.6%) in the FAPD group (*P* = 0.004).

**Table 2:** Comparison of alarm symptoms between the two groups of patients with organic disease and FAPD.

Alarm symptoms	Organic disease (*n* = 224)		FAPD (*n* = 347)		*χ* ^2^	*P*-value	OR (95% CI)
	Yes (*n*, %)	No (*n*, %)	Yes (*n*, %)	No (*n*, %)			
Fever	24 (10.7)	200 (89.3)	0 (0)	347 (100)	38.81	**0**.**000**	NA
Diarrhea	45 (20.1)	179 (79.9)	15 (4.3)	332 (95.7)	35.98	**0**.**000**	5.56 (3.02–10.26)
Constipation	14 (6.3)	210 (93.7)	17 (4.9)	330 (95.1)	0.48	0.487	1.29 (0.63–2.68)
Vomiting	54 (24.1)	170 (75.9)	29 (8.4)	318 (91.6)	27.18	**0**.**000**	3.48 (2.14–5.68)
Bloody stool	47 (21.0)	177 (79.0)	7 (2.0)	340 (98,0)	57.18	**0**.**000**	12.90 (5.71–29.73)
Abdominal distention	18 (8.0)	206 (92.0)	12 (3.5)	335 (96.5)	5.73	**0**.**02**	2.44 (1.15–5.17)
Perianal lesions	13 (5.8)	211 (94.2)	1 (99.7)	346 (0.3)	17.31	**0**.**000**	21.32 (2.77–164.13)
Retardation of growth	10 (4.5)	214 (95.5)	12 (3.5)	335 (96.5)	NA	**0**.**005F**	7.83 (1.70–36.07)
Weight loss	69 (30.8)	155 (69.2)	11 (3.2)	336 (96.8)	86.28	**0**.**000**	13.60 (7.00–26.42)
Delayed puberty	2 (0.9)	222 (99.1)	0 (0)	347 (100)	NA	0.153F	NA
Oral ulcer	7 (3.1)	217 (96.9)	0 (0)	347 (100)	NA	**0**.**001F**	NA
Family History	59 (26.3)	165 (73.7)	15 (4.3)	332 (95.7)	58.50	**0**.**000**	7.91 (4.36–14.37)

FAPD, functional abdominal pain disorder; CI, confidence interval; NA, not applicable; F, Fisher's exact test.

*P*-value of <0.05 was considered statistically significant.

Laboratory index comparisons are presented in Table 3, with odds ratios (ORs) reported for continuous variables representing the odds of having an abnormal value vs. a normal value, based on standard pediatric clinical cutoff thresholds. The WBC, PLT, CRP, and ESR levels were significantly higher in the organic disease group than those in the FAPD group (*P* < 0.01). A total of 31 children had functional constipation (FC), with a FC positivity rate of 19.4%, which was higher than that in the non-constipated FAPD children (13.8%), although the difference was not statistically significant (*P* = 0.09).

**Table 3:** Comparison of laboratory indices in the two groups of patients with organic diseases and FAPD $\left(\bar{x}\pm s\right)$.

Laboratory indicators	Organic matter (*n* = 224)	Functionality (*n* = 347)	*t*/*χ*^2^	*P*-value	OR (95% CI)
WBC (×10^9^/L)	7.68 ± 3.65	6.81 ± 2.06	−3.62	**0**.**000**	
WBC (A/N)	45/175	38/309	9.73	**0**.**002**	2.09 (1.31–3.35)
PLT (×10^12^/L)	327.18 ± 108.77	288.12 ± 62.47	−5.44	**0**.**000**	
PLT (A/N)	122/102	132/215	14.87	**0**.**000**	1.95 (1.39–2.74)
CRP (mg/L)	10.32 ± 14.13	6.93 ± 3.58	−4.27	**0**.**000**	
CRP (A/N)	30/194	3/344	39.24	**0**.**000**	17.73 (5.34–58.86)
ESR (mm/h)	18.29 ± 23.05	10.15 ± 16.16	−6.06	**0**.**000**	
ESR (A/N)	55/169	39/308	17.55	**0**.**000**	2.57 (1.64–2.04)
Fecal FC (A/N)	130/94	45/302	130.08	**0**.**000**	9.28 (6.16–14.00)
Fecal OB (A/N)	88/136	8/339	133.10	**0**.**000**	27.42 (12.94–58.08)

A/N, abnormal/normal; WBC, white blood cell count; PLT, platelet count; CRP, C-reactive protein; ESR, erythrocyte sedimentation rate; FC, fecal calprotectin; OB, fecal occult blood; OR, odds ratio; CI, confidence interval.

*P*-value of <0.05 was considered statistically significant.

### 3.3 Laboratory findings

Table 3 shows the comparisons of laboratory indices. The levels of white blood cells (WBC), platelets (PLT), CRP, and ESR were significantly higher in children with organic disease than those in children with FAPD (*P* < 0.01). The organic disease group had a significantly higher frequency of positive fecal and blood inflammatory indices (*P* < 0.01).

Among the 31 children diagnosed with functional constipation, the FC positivity rate was 19.4%, slightly higher than that in non-constipated children with FAPD (13.8%), without statistical significance (*P* = 0.09).

**Figure 2:**
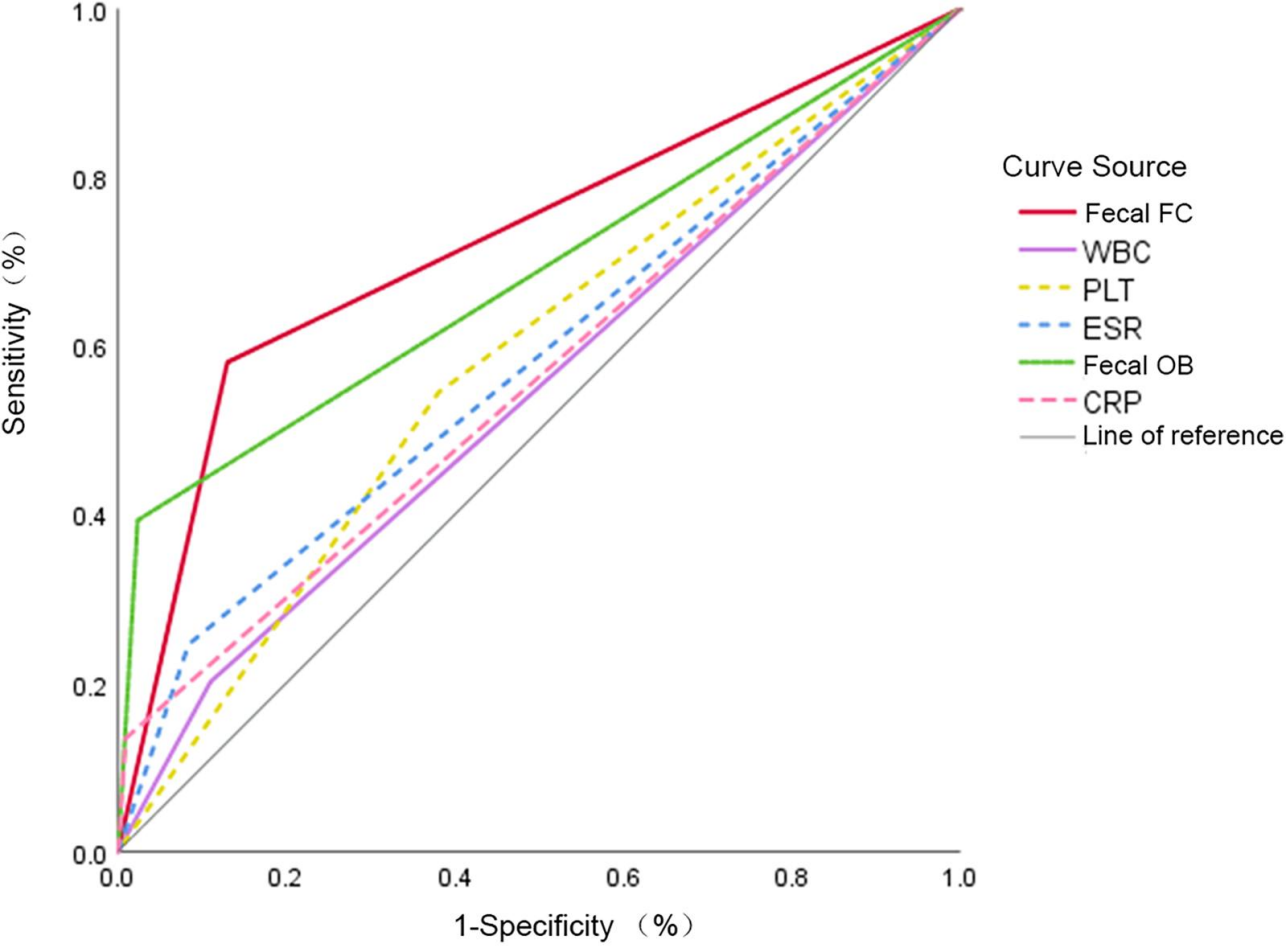
ROC curves for each laboratory indicator. This figure provides the ROC curves for the various laboratory parameters (WBC, PLT, CRP, ESR, OB, fecal FC) analyzed to assess their diagnostic effectiveness in separating organic disease from the FAPD group.

Figure 2 illustrates the ROC curves of each diagnostic indicator. The derived AUC, sensitivity, and specificity values for the respective indicators are presented in Table 4. FC had the highest diagnostic accuracy (AUC = 0.725; sensitivity = 58%; specificity = 87%) among the individual markers of WBC, PLT, CRP, ESR, and fecal OB (*P* < 0.01).

**Table 4:** AUC and diagnostic efficacy of each laboratory indicator and co-diagnostic indicators.

Diagnostic indicators	A/N	AUC	95% CI	Sen, %	Sep, %	PPV, %	NPV, %
Alarm symptoms	227/344	0.729	0.68–0.77	70.5	80.1	69.6	80.8
Blood indicators	308/263	0.630	0.58–0.68	63.4	52.2	45.5	68.8
Fecal indicators	203/368	0.787	0.75–0.83	67.9	85.3	74.9	80.4
Alarm symptoms + blood indicators	450/121	0.750	0.71–0.79	96.0	32.3	47.8	92.6
Alarm symptoms + fecal indicators	306/265	0.841	0.81–0.88	90.6	70.3	66.3	92.1
Alarm symptoms + blood + feces	470/101	0.834	0.80–0.87	99.1	28.5	39.2	98.0
Fecal FC	175/396	0.725	0.68–0.77	58.0	87.0	74.3	76.3
Fecal OB	96/475	0.685	0.64–0.73	39.3	97.7	91.7	71.4
WBC	83/488	0.546	0.50–0.60	20.1	89.0	54.2	63.3
PLT	254/317	0.582	0.53–0.63	54.5	62.0	48.0	67.8
CRP	33/538	0.563	0.51–0.61	13.4	99.1	90.9	63.9
ESR	84/487	0.581	0.53–0.63	24.6	91.6	65.5	65.3

A/N, abnormal/normal; AUC, area under the ROC curve; CI, confidence interval; Sen, sensitivity; Spec, specificity; PPV, positive predictive value; NPV. negative predictive value; FC, fecal calprotectin; OB, fecal occult blood; WBC, white blood cell count; PLT, platelet count; CRP, C-reactive protein.

The term “calreticulin” discussed previously refers to an endoplasmic reticulum protein that has been investigated in other studies as an inflammatory marker. FC was the only biomarker of intestinal inflammation that was used in this present study.

### 3.4 ROC curves and AUC for six diagnostic strategies

The ROC curve analyses for the six defined diagnostic strategies are shown in Figure 1 and summarized in Table 4. Strategy 5 (alarm symptoms + fecal indicators) displayed the best overall diagnostic information with an AUC of 0.841, a sensitivity of 90.6%, and a specificity of 70.3%, which outperformed all models.

Although Strategy 6 (alarm symptoms + blood + fecal indicators) had slightly higher sensitivity (99.1%) than Strategy 5, specificity and PPV were still lower than Strategy 5. These results support the notion that alarm symptoms in combination with fecal markers are the most effective and least invasive way to differentiate organic from FAP in children.

### 3.5 ROC curves and AUC for six diagnostic strategies

Figure 1 and Table 4 present ROC curve comparisons for the six predefined strategies. Strategy 5 (alarm symptoms + fecal indices**)** achieved the highest diagnostic performance with AUC = 0.841, sensitivity = 90.6%, and specificity = 70.3%, outperforming all other approaches. Although Strategy 6 (alarm + blood + fecal indices) yielded slightly higher sensitivity (99.1%), its specificity and PPV were lower.

*The AUC values are annotated within Figures 1 and 2 for clarity.*

## 4 Discussion

CAP in children and adolescents is often reported as a source of significant anxiety for patients and their parents. Although many cases are functional, some pediatric patients have an underlying organic gastrointestinal disease (8). Endoscopy is an important procedure in the diagnosis of IBD and other organic diseases. According to the revised criteria, it is recommended that alarm symptoms are present or inflammatory markers are elevated, including FC (11). However, endoscopy requires bowel preparation, anesthesia, and hospitalization, leading to procedural and financial burdens. Thus, non-invasive diagnostic tools with high sensitivity and specificity can be valuable for early screening (12).

CRP, ESR, and PLT are traditional inflammatory markers used to assess disease activity in IBD; however, their accuracy to diagnose and differentiate organic conditions from functional ones is limited (13). In our study, AUC values of individual blood markers (WBC, PLT, CRP, and ESR) did not exceed 0.6, which indicates poor discriminatory power. While PLT was more sensitive than the other indices, its performance was still not optimal. The underlying cause of thrombocytopenia in some cases remains unclear, but it may be related to bone marrow suppression or inflammatory cytokine consumption; nonetheless, this observation is non-specific and potentially misleading.

It should be noted that all analyses in the present study were based solely on FC, and no serum calreticulin testing was performed. Calprotectin is a 36 kDa calcium-binding protein that is present in high quantities in neutrophils; its concentration in feces is directly proportional to the degree of inflammation at the intestinal mucosa (14). The clinical studies continually demonstrated the usefulness of FC as an indicator of intestinal inflammation and its sensitivity as a biomarker when compared with systemic markers, including CRP and the ESR (15). In other words, while elevated FC is not limited to IBD, it may also be observed in bacterial enteritis, gastritis, polyps in the colon, diverticulitis, and NSAID-induced injuries to the mucosa (16). For that reason, FC serves well as a non-invasive method for differentiating between functional and organic causes, but is not a single disease-specific indicator.

In this investigation, we employed a uniform FC cutoff of 60 µg/g for simplicity and also to make comparisons to previous pediatric studies. However, stratifying age results demonstrated that younger children had a higher FC positivity rate, indicating that differences might exist due to physiological changes and variability around specific gravity. Our secondary analysis using a threshold of 100 µg/g increased specificity modestly without decreasing sensitivity, suggesting there may be clinical benefit in considering alternative FC cutoffs.

Although blood tests such as CRP, ESR, and platelet count are traditionally included in CAP evaluation, our findings show that their diagnostic accuracy is substantially lower than that of fecal markers. Therefore, routine blood testing may be unnecessary in children without alarm symptoms, and FC/OB testing can serve as an initial, less invasive screening tool.

## 5 Clinical implications of mildly elevated FC

In terms of clinical decision-making, when FC levels are mildly elevated (60–100 µg/g) without alarm-type symptoms, repeat testing for FC may serve well without proceeding to immediate endoscopy. This could prevent unnecessary invasive procedures in younger children, which also remains in line with stepwise recommendations; meanwhile, mild elevation of FC could also present in children with functional constipation due to persistent low-grade mucosal immune activation, which might reduce FC specificity but not diagnostic value when evaluated in context.

Our results coincide with the recommendations outlined in the 2021 ESPGHAN position paper which recommends FC be employed as the first-line, differential test to distinguish between inflammatory and functional gastrointestinal disorders (9). Additionally, Zhu et al. (10) concluded that stool testing combining fecal hemoglobin and FC improved the diagnostic performance and recommendations overall for concluding significant bowel disease. This also further legitimizes stool-based fecal testing as a reasonable front-line diagnostic tool in pediatric gastroenterology.

Fecal OB testing remains a reliable orthogonal measurement, provided that patients subject themselves to a relatively standardized time period of dietary restrictions; however, it is susceptible to a history of false-positive interference influenced by dietary habits (17, 18). In the context of this study, it is worth noting that the combined diagnostic value of fecal FC with OB was superior to any of the individual blood markers, all combined, and this addendum reinforces that stool-based indicators are better indicators of ongoing inflammation than blood (20).

Alarm symptoms appeared and were strongly associated particularly with vomiting, weight loss, diarrhea, and bloody stool, which are highly indicative of organic pathology and aligned with other evidence (19, 20). Furthermore, the combination of strategies evaluating all put forward diagnostic combinations—Strategy 5 combination achieved the best diagnostic performance (AUC = 0.841), showing fecal-based indices, including FC and OB, added significant value beyond the blood based-pointing toward inflammation or pathology.

## 6 Limitations

There are several limitations to this study. First, although it was a retrospective design, patient allocation may have been biased. Second, not all in the FAPD group received a complete endoscopic examination, which may partly have led to verification bias. Third, the use of a single FC cutoff for all ages may have affected classification accuracy—especially in younger children. Fourth, despite dietary modifications prior to sample collection, there is still the possibility of a residual false-positive OB result. Fifth, we did not measure serum inflammatory biomarkers (such as calreticulin or other systemic markers), which limited a cross-validation of mucosal and systemic inflammation. Finally, as a single-center study, there may be challenges to generalizability. Therefore, we conclude that future multicenter, prospective trials are warranted to assess age-specific reference values ​​and standardized diagnostic pathways.

## 7 Conclusion

Fecal markers combined with alarm symptoms provide a simple, accurate, and non-invasive approach for distinguishing organic from FAP in children. This method reduces unnecessary endoscopic or blood testing while maintaining diagnostic precision. It is particularly suitable for use in primary and secondary healthcare settings. Future diagnostic strategies should incorporate age-adjusted FC cutoffs, interpret mild FC elevations cautiously, and ensure proper preparation before OB testing. Screening for extraintestinal symptoms, such as arthritis and arthralgia, may further enhance early detection of systemic disease. A stepwise approach using alarm symptoms and fecal markers before invasive testing should be integrated into pediatric gastroenterology practice. Non-invasive strategies using alarm symptoms with fecal markers may reduce unnecessary blood testing and endoscopy, especially when FC values are only mildly elevated.

## Data Availability

The raw data supporting the conclusions of this article will be made available by the authors, without undue reservation.
